# Accuracy and Clinical Significance of Intraoperative Gross Extrathyroidal Extension (T3b) Assessment in Differentiated Thyroid Carcinoma

**DOI:** 10.3390/cancers17243914

**Published:** 2025-12-07

**Authors:** Solji An, Joonseon Park, Kwangsoon Kim, Ja Seong Bae

**Affiliations:** Department of Surgery, College of Medicine, The Catholic University of Korea, Seoul 06591, Republic of Korea; yogurt2436@gmail.com (S.A.); joonsunny@naver.com (J.P.); jaseong@gmail.com (J.S.B.)

**Keywords:** thyroid carcinoma, thyroidectomy, extrathyroidal extension, strap muscle, intraoperative assessment, recurrence

## Abstract

Objective: In the eighth edition of the American Joint Committee on Cancer tumor–node–metastasis staging system, gross extrathyroidal extension (ETE) into the strap muscles is classified as T3b when identified during surgery. In clinical practice, this invasion is primarily assessed intraoperatively by the surgeon and documented in the operative report, forming the basis of the final T3b staging. Because this evaluation is inherently subjective, its diagnostic accuracy remains uncertain. This study evaluated the accuracy of intraoperative gross ETE assessment and whether misclassification affects recurrence outcomes. Methods: In total, 4987 patients who underwent thyroidectomy at Seoul St. Mary’s Hospital during 2017–2022 were analyzed. Patients were categorized by concordance between intraoperative findings and final pathology: confirmed gross ETE (Group A), intraoperative overestimation (Group B), and intraoperative underestimation (Group C). Clinical characteristics, recurrence rates, and predictors of inaccurate assessment were compared. Results: Of the cohort, 179 patients (3.6%) were judged intraoperatively to have gross ETE, classified as Group A (141 patients), Group B (38), and Group C (33). Recurrence rates were not significantly different among groups (6.4%, 2.6%, and 3.0% in Groups A, B, and C, respectively). Other than lymphatic invasion and tumor size, baseline characteristics were comparable among groups. Multivariate analysis identified age (odds ratio [OR]: 0.961; 95% confidence interval [CI]: 0.932–0.990; *p* = 0.009), tumor location (OR: 0.182; 95% CI: 0.056–0.591; *p* = 0.005), and lymphatic invasion (OR: 0.292; 95% CI: 0.118–0.719; *p* = 0.007) as independent predictors of inaccurate intraoperative evaluation. Conclusions: Among 179 patients suspected of gross ETE intraoperatively, 21.2% showed no muscle invasion on pathology. Although recurrence rates were similar across groups, recurrence-free survival tended to be lower in Group A relative to Group B, indicating the potential prognostic relevance of accurate intraoperative T3b identification. Long-term follow-up is needed to confirm this trend.

## Simple Summary

In thyroid cancer, assessing whether the tumor invades the strap muscles (T3b) is critical for accurate staging and postoperative management. Unlike most cancers, this evaluation is performed visually by surgeons during the operation. However, the accuracy of this intraoperative judgment compared with pathological findings remains uncertain. In this study of 4987 patients who underwent thyroidectomy during 2017–2022, we compared surgeons’ impressions of gross extrathyroidal extension with final pathology and analyzed recurrence outcomes. We found that 21% of cases considered muscle-invasive during surgery showed no invasion under microscopic examination. Although recurrence rates were similar, accurate intraoperative identification was associated with slightly lower recurrence-free survival. These findings suggest that intraoperative judgment of gross invasion may have prognostic relevance, underscoring the need for careful evaluation and long-term follow-up.

## 1. Introduction

Although differentiated thyroid cancer (DTC) is associated with favorable prognosis and low mortality [1,2,3,4,5], multiple clinicopathologic factors cause variations in survival outcomes [5,6,7]. To address these differences, the American Joint Committee on Cancer/Union for International Cancer Control (AJCC/UICC) introduced the tumor–node–metastasis (TNM) staging system to predict prognosis and guide management strategies [1,8,9]. The eighth edition, the most recent version published in 2016, introduced major revisions relative to the seventh edition, including the addition of the T3b category [1,8,9,10].

In the seventh edition, microscopic extrathyroidal extension (ETE) detected on pathology was classified as T3 disease, often resulting in overstaging with limited impact on recurrence and survival [8,11,12,13,14,15,16]. Therefore, the eighth edition revised the T3 category, restricting T3b to cases in which gross extension to the strap muscles is identified intraoperatively, with this determination relying on the surgeon’s direct assessment during the procedure [8,10,12].

Notably, intraoperative evaluation of gross ETE to the strap muscles is subjective [1,17] and can be affected by surgeon experience, operative visibility, and tumor location, as well as the presence of fibrosis or thyroiditis, resulting in interobserver variation and possible misclassification [18,19]. Moreover, the prognostic value of T3b remains debated. Although studies have reported poorer outcomes in patients with gross strap muscle invasion, others have noted no significant differences when complete resection is achieved [6,8,9,13,14,20,21,22,23]. However, evidence supporting the clinical implications of cases where T3b is over- or under-estimated intraoperatively remains limited [3,18].

The present study evaluated the accuracy of intraoperative assessment of gross ETE to the strap muscles relative to pathological confirmation, identified clinical factors associated with misjudgment, and investigated whether such misclassification influences recurrence and long-term prognosis in patients with DTC. To our knowledge, this study is among the first to provide systematic evaluations of surgeon-based T3b assessment accuracy and report its prognostic relevance in a large institutional cohort.

## 2. Materials and Methods

### 2.1. Patients

This study was conducted between January 2017 and December 2022 at Seoul St. Mary’s Hospital (Seoul, Republic of Korea), a high-volume endocrine surgery center performing >800 thyroidectomies annually, and included 4987 patients who underwent thyroidectomy. Of these patients, 945 who received surgery for nonmalignant indications (such as Graves’ disease or benign nodules), were lost to follow-up, or had missing data were excluded from the final analysis, with 4042 patients included. Patients were categorized according to the presence of gross ETE to the strap muscles during surgery and muscle invasion on final histopathology.

**Figure 1:**
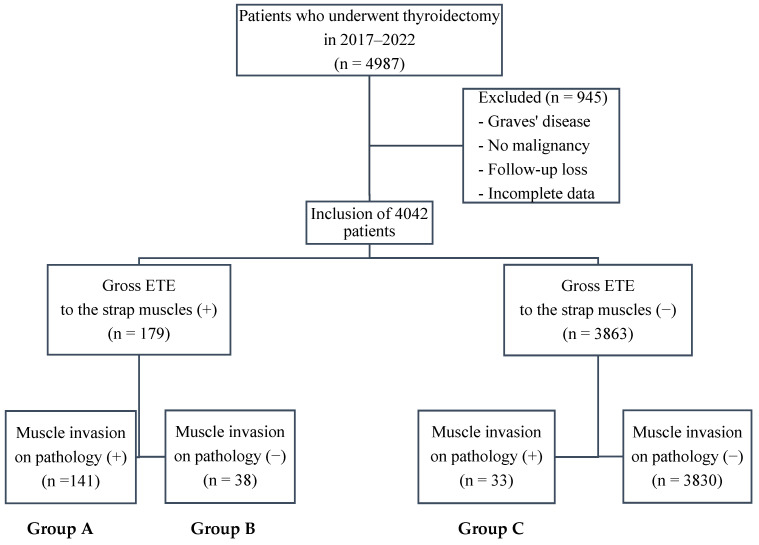
Flowchart of patient grouping. Patients were stratified by intraoperative assessment of gross extrathyroidal extension (ETE) to the strap muscles as well as the presence of muscle invasion on final pathology. Group A: intraoperative gross ETE (+) and pathology (+); Group B: intraoperative gross ETE (+) and pathology (−); Group C: intraoperative gross ETE (−) and pathology (+). Abbreviations: ETE, extrathyroidal extension.

Group A comprised patients who had gross ETE to the strap muscles identified intraoperatively and confirmed as gross muscle invasion on final pathology (*n* = 141). Group B included patients suspected intraoperatively of gross ETE but without gross muscle invasion on pathology (*n* = 38). Group C contained patients with no intraoperative suspicion of gross ETE but with gross muscle invasion on pathology (*n* = 33) (Figure 1).

The clinicopathological characteristics and outcomes of patients in Groups A, B, and C were compared. In cases with multifocal tumors, the size and location of the lesion judged to have muscle invasion were used for analysis. TNM staging followed the eighth edition of the AJCC/UICC TNM system [10]. Gross ETE to the strap muscles was evaluated visually during surgery, and the tumor location was extracted from surgeons’ operative notes. Specifically, “misjudgment” was defined as overestimation, referring to cases where gross ETE was suspected intraoperatively despite no muscle invasion being detected on final pathology. All patients underwent surgery and postoperative treatment according to the 2015 American Thyroid Association management guidelines for DTC [24].

Postoperatively, all patients were monitored regularly by testing for serum thyroid function, thyroglobulin content, and anti-thyroglobulin antibody levels as well as via neck ultrasonography. For patients with suspected recurrence or metastasis during follow-up, additional imaging, such as computed tomography, positron emission tomography/computed tomography, or radioactive iodine whole-body scanning, was performed or biopsy was used to confirm recurrence or metastasis. The median follow-up duration was 44.0 ± 17.4 months (range: 16–83 months).

This study adhered to the Declaration of Helsinki (revised in 2013) [25], and was approved by the Institutional Review Board of Seoul St. Mary’s Hospital, The Catholic University of Korea (IRB No: KC23RISI0664). The need for informed consent was waived owing to the retrospective study design.

### 2.2. Statistical Analysis

Continuous variables were analyzed using Student’s *t*-test and presented as means ± standard deviations. Categorical variables were analyzed using Pearson’s chi-square test or Fisher’s exact test and expressed as counts and percentages. Univariate and multivariate logistic regression analyses were used to identify clinical factors associated with misjudgment of ETE (Group A vs. Group B), with hazard ratios (HRs) and 95% confidence intervals (CIs) calculated. The multivariate logistic regression model included age, sex, palpation, thyroiditis, tumor location, tumor size, multifocality, lymphatic invasion, harvested lymph nodes (LNs), positive LNs, and N stage as covariates. Recurrence-free survival (RFS) was compared across groups using Kaplan–Meier survival curves. *p* < 0.05 was considered statistically significant. All analyses were conducted using the Statistical Package for the Social Sciences software v24.0 (IBM Corp., Armonk, NY, USA).

## 3. Results

### 3.1. Comparison of Baseline Clinicopathological Characteristics

Table 1, Table 2 and Table 3 summarize the baseline clinicopathological characteristics across Groups A–C. Between Groups A and B (Table 1), Group A exhibited a significantly larger tumor size (2.0 ± 1.0 vs. 1.4 ± 1.0 cm, *p* = 0.005) and a higher frequency of lymphatic invasion (69.5% vs. 47.4%, *p* = 0.011). Other than these differences, including recurrence cases, no variables differed significantly between the two groups.

Between Groups B and C (Table 2), Group C exhibited significantly larger tumors (1.4 ± 1.0 vs. 2.2 ± 1.5 cm, *p* = 0.016), a higher incidence of vascular invasion (0% vs. 12.1%, *p* = 0.042), and more frequent lymphatic invasion (47.4% vs. 72.7%, *p* = 0.030). Other characteristics, including recurrence cases, did not differ significantly between the groups.

Finally, comparison between Groups A and C (Table 3) revealed no statistically significant differences in any clinicopathological characteristics.

### 3.2. Analyses of Clinical Factors Influencing the Misjudgment of ETE

To identify factors associated with the misjudgment of gross ETE, univariate and multivariate logistic regression analyses were performed using data from Groups A and B (Table 4). In the multivariate model, younger age (odds ratio [OR], 0.961; 95% CI, 0.932–0.990; *p* = 0.009), mid-portion tumor location (OR, 0.182; 95% CI, 0.056–0.591; *p* = 0.005), and absence of lymphatic invasion (OR, 0.292; 95% CI, 0.118–0.719; *p* = 0.007) were independently associated with the misjudgment of gross ETE.

### 3.3. Analysis of RFS and Recurrence Events

**Figure 2:**
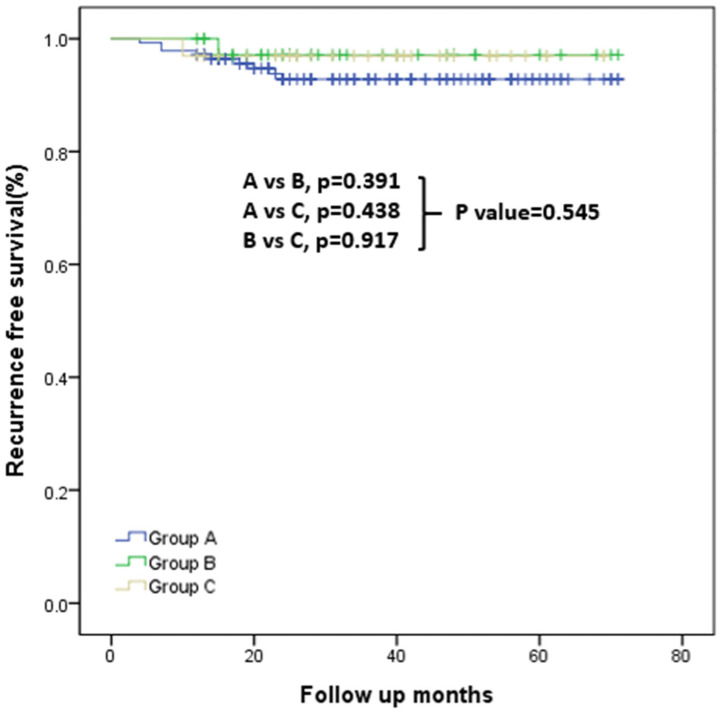
Kaplan–Meier curves for recurrence-free survival in Groups A, B, and C. Overall comparison: *p* = 0.545. Pairwise comparisons: Group A vs. Group B, *p* = 0.391; Group A vs. Group B, *p* = 0.438; Group B vs. Group C, *p* = 0.917.

During a median follow-up of 44.0 ± 17.4 months (range: 16–83 months), RFS did not differ significantly among Groups A–C based on Kaplan–Meier survival curve analysis (Figure 2). Details of recurrence cases are provided in Supplementary Table S1.

## 4. Discussion

DTC accounts for most thyroid cancers and is associated with a high survival rate but frequent recurrence and metastasis [2,3,8]. Because patients with DTC typically show long-term survival, accurate assessment of recurrence risk is essential [8]. In this context, the AJCC/UICC TNM staging system has been widely used globally to predict survival and recurrence in DTC cases [8,10]. In the eighth edition, a major revision was the introduction of T3b [6,8], defined as gross invasion of the strap muscles regardless of tumor size. Importantly, the surgeon’s intraoperative impression of gross ETE, recorded during surgery, is incorporated directly into T3b classification [10].

Since the adoption of the TNM staging system’s eighth edition, several studies have investigated whether T3b meaningfully affects clinical outcomes. Xu et al. reported poorer overall and cancer-specific survival among patients with T3b, particularly when tumors were ≥2 cm or patient age was ≥55 years [6]. Xiang et al. also reported T3b’s association with worse cancer-specific survival in a SEER-based analysis [26]. Conversely, multiple studies assessing T3b and survival have reported no significant survival differences when invasion is limited to the strap muscles [8,9,13,20,22,23]. Despite these discrepancies in survival outcomes, most studies consistently demonstrate a strong association between T3b and recurrence [6,9,13,20,21].

Given this relevance to recurrence, precise assessment of T3b is critical. However, because T3b relies on subjective intraoperative assessment, its accuracy assessed in real-world practice remains uncertain. In our cohort, 38 of 179 patients (21.2%) initially judged to have gross ETE to the strap muscles showed no muscle invasion on final pathology, a higher proportion than the 16 of 145 cases (11%) reported by Jung et al. [18]. In our study, the prediction performance showed a sensitivity of 81%, specificity of 99%, positive predictive value of 78.8%, and negative predictive value of 99.1%. The relatively low positive predictive value reflects a tendency toward overestimation of gross ETE intraoperatively, whereas the high specificity and negative predictive value indicate that the absence of gross ETE is assessed with high reliability.

Consistent with prior studies [3,6,9,20], both Groups A and C, including patients with pathologically proven muscle invasion, had larger tumors compared with Group B (Table 1, Table 2 and Table 3). Previous research has also shown that even minimal ETE (as in Group C) is associated with increased tumor size relative to cases without ETE [27,28,29]. Jung et al. similarly reported that misclassified cases (analogous to Group B) tended to have smaller tumors compared with other cases [18].

To further examine misclassification, we analyzed clinicopathologic factors associated with Group B (Table 4). Younger patient age was correlated with a higher likelihood of misclassification. Additionally, tumors located in the upper rather than mid portion as well the absence of lymphatic invasion were significantly associated with incorrect intraoperative assessment of gross ETE to the strap muscles.

Sahin et al. postulated that younger patients may have a higher incidence of interstitial fibrosis, testing this using a cutoff age of 45 years; indeed, they observed a higher proportion of interstitial fibrosis cases in patients aged <45 years, although the difference was not statistically significant [30]. These findings may contribute to inaccurate judgments of gross ETE by blurring the boundary between the tumor and surrounding tissues, thereby reducing the reliability of intraoperative evaluation. Further research is warranted to clarify this mechanism. Kuo et al. also investigated factors predicting ETE preoperatively and reported that older age was linked to a higher likelihood of ETE, suggesting that age-related tumor microenvironmental changes may promote invasion [3]. Considering their data alongside our results, surgeons should be particularly cautious to avoid overestimating gross ETE in younger patients.

Several studies have shown that tumors in the upper thyroid pole are more likely to be malignant compared with those in the lower pole [19,31,32,33]. This elevated risk has been attributed to the tortuous venous drainage of the upper pole, which may promote accumulation of reactive oxygen species (ROS) [31,32,33]. ROS accumulation has also been associated with fibrosis and adhesion to adjacent structures [34,35]. Based on these findings, we hypothesize that the higher ROS burden in the thyroid’s upper pole promotes fibrosis and adhesion, contributing to overestimation of muscle invasion during intraoperative assessment. Additionally, upper pole tumors more frequently exhibit capsular invasion [19], and dissection of the upper pole and cricothyroid space is often constrained by the overlying sternothyroid muscle, which restricts the surgical field of view [36]. Therefore, meticulous evaluation is required when assessing gross ETE to the strap muscles in upper pole tumors.

Gan et al. reported that Hashimoto thyroiditis can complicate surgical dissection due to tissue adhesion [37]. Jung et al. suggested that parenchymal abnormalities, including inflammation or thyroiditis, may predispose surgeons to interpret dense adhesions as gross ETE [18]. They further proposed that histological changes induced by fine-needle aspiration (FNA), such as hemorrhage, granulation tissue formation, fibrosis, and capsular pseudoinvasion, could contribute to diagnostic inaccuracies [18,38]. Although neither thyroiditis nor FNA-induced changes reached statistical significance in these studies, and our cohort also showed no significant association with thyroiditis, further research is needed to clarify these potential contributors [18].

Comparison of RFS across the three groups revealed a trend toward lower RFS in Group A, although differences were not statistically significant (Figure 2). This pattern suggests that accurate intraoperative identification of gross ETE may have clinical relevance; however, the numerically lower RFS in Group A could also reflect underlying tumor aggressiveness and should be interpreted cautiously.

To minimize intersurgeon variability, adopting standardized intraoperative criteria or photographic documentation of gross ETE could enhance consistency and objectivity in T3b assessment. The development of clear intraoperative guidelines, potentially combined with preoperative imaging and multidisciplinary review, may further improve the reliability of surgeon-based staging in DTC.

This study has several limitations. First, the lack of statistically significant differences in RFS may reflect the relatively short follow-up period, highlighting the need for longer-term assessment of recurrence and survival. Second, intraoperative judgments were made by four different surgeons, and individual experience may have introduced interobserver variability [18]. Third, the smaller sample sizes of Groups B and C relative to Group A may have reduced the statistical stability of our analyses. These imbalances could yield imprecise or biologically inconsistent ORs and limit the reliability of subgroup comparisons. Finally, the retrospective, single-center design may have introduced selection and information biases.

## 5. Conclusions

This study evaluated the accuracy of intraoperative T3b assessment and revealed that 21.2% of cases were misjudged. Factors associated with misclassification included younger age, upper pole tumor location, and absence of lymphatic invasion. Although recurrence rates did not differ significantly among the three groups, Group A, in which gross ETE was accurately identified, tended to show lower RFS relative to Group B, in which gross ETE was overestimated. These findings underscore the need for standardized criteria for intraoperative gross ETE assessment to avoid overstaging and unnecessary adjuvant therapy. Further studies with extended follow-up are required to validate the current findings.

## Figures and Tables

**Table 1:** Baseline clinicopathological characteristics between Groups A and B.

	Group A (*n* = 141)	Group B (*n* = 38)	*p*-Value
Age (years)	52.2 ± 14.9 (range, 17–84)	47.4 ± 14.5 (range, 20–81)	0.760
Male	38 (27.0%)	9 (23.7%)	0.685
Palpation	34 (24.1%)	7 (18.4%)	0.459
Extent of surgery			
Total	86 (61.0%)	20 (52.6%)	0.352
Less than total	55 (39.0%)	18 (47.4%)	
CLND	141 (100%)	37 (97.4%)	0.212
LND	40 (28.4%)	8 (21.1%)	0.366
Thyroiditis	53 (37.6%)	14 (36.8%)	0.933
Pathology			
PTC	138 (97.9%)	38 (100%)	1
PDTC	3 (2.1%)	0	
Tumor location			
Upper	8 (5.7%)	9 (23.7%)	0.222
Mid	113 (80.1%)	21 (55.3%)	
Lower	20 (14.2%)	8 (21.0%)	
Tumor size (cm)	2.0 ± 1.0 (range, 0.4–5.8)	1.4 ± 1.0 (range, 0.3–5.8)	0.005
Multifocality			
Unilateral	24 (17.0%)	11 (28.9%)	0.863
Bilateral	18 (12.8%)	3 (7.9%)	
Vascular invasion	12 (8.5%)	0	0.073
Lymphatic invasion	98 (69.5%)	18 (47.4%)	0.011
Perineural invasion	8 (5.7%)	0	0.206
Harvested LNs	25.9 ± 31.2 (range, 0–164)	20.3 ± 20.5 (range, 1–80)	0.186
Positive LNs	6.2 ± 7.7 (range, 0–33)	5.3 ± 7.1 (range, 0–25)	0.553
N stage			
N0	40 (28.4%)	13 (34.2%)	0.385
N1a	66 (46.8%)	18 (47.4%)	
N1b	35 (24.8%)	7 (18.4%)	
RAI ablation	62 (44.0%)	14 (36.8%)	0.430
RAI mean dose (mCi)	111.3 ± 46.5	104.2 ± 28.5	0.590
Recurrence	9 (6.4%)	1 (2.6%)	0.691

Data are expressed as patient counts (%) or means ± standard deviations. Abbreviations: CLND, central lymph node dissection; LND, lateral neck dissection; PTC, papillary thyroid carcinoma; PDTC, poorly differentiated thyroid carcinoma; LN, lymph node; N, node; RAI, radioactive iodine.

**Table 2:** Baseline clinicopathological characteristics between Groups B and C.

	Group B (*n* = 38)	Group C (*n* = 33)	*p*-Value
Age (years)	47.4 ± 14.5 (range, 20–81)	49.4 ± 14.9 (range, 18–76)	0.562
Male	9 (23.7%)	7 (21.2%)	0.804
Palpation	7 (18.4%)	5 (15.2%)	0.714
Extent of surgery			
Total	20 (52.6%)	19 (57.6%)	0.676
Less than total	18 (47.4%)	14 (42.4%)	
CLND	37 (97.4%)	32 (97.0%)	1
LND	8 (21.1%)	9 (27.3%)	0.540
Thyroiditis	14 (36.8%)	10 (30.3%)	0.561
Pathology			
PTC	38 (100%)	32 (97.0%)	0.465
PDTC	0	1 (3.0%)	
Tumor location			
Upper	9 (23.7%)	3 (9.1%)	0.157
Mid	21 (55.3%)	25 (75.8%)	
Lower	8 (21.0%)	5 (15.2%)	
Tumor size (cm)	1.4 ± 1.0 (range, 0.3–5.8)	2.2 ± 1.5 (range, 0.5–6.0)	0.016
Multifocality			
Unilateral	11 (28.9%)	7 (21.2%)	0.255
Bilateral	3 (7.9%)	7 (21.2%)	
Vascular invasion	0	4 (12.1%)	0.042
Lymphatic invasion	18 (47.4%)	24 (72.7%)	0.030
Perineural invasion	0	1 (3.0%)	0.465
Harvested LNs	20.3 ± 20.5 (range, 1–80)	25.7 ± 30.4 (range, 0–108)	0.384
Positive LNs	5.3 ± 7.1 (range, 0–25)	6.0 ± 6.2 (range, 0–22)	0.692
N stage			
N0	13 (34.2%)	15 (45.5%)	0.564
N1a	18 (47.4%)	10 (30.3%)	
N1b	7 (18.4%)	8 (24.2%)	
RAI ablation	14 (36.8%)	17 (51.5%)	0.214
RAI mean dose (mCi)	104.3 ± 28.5	117.6 ± 52.9	0.403
Recurrence	1 (2.6%)	1 (3.0%)	1

Data are expressed as patient counts (%) or means ± standard deviations. Abbreviations: CLND, central lymph node dissection; LND, lateral neck dissection; PTC, papillary thyroid carcinoma; PDTC, poorly differentiated thyroid carcinoma; LN, lymph node; N, node; RAI, radioactive iodine.

**Table 3:** Baseline clinicopathological characteristics between Groups A and C.

	Group A (*n* = 141)	Group C (*n* = 33)	*p*-Value
Age (years)	52.2 ± 14.9 (range, 17–84)	49.4 ± 14.9 (range, 18–76)	0.334
Male	38 (27.0%)	7 (21.2%)	0.498
Palpation	34 (24.1%)	5 (15.2%)	0.266
Extent of surgery			0.718
Total	86 (61.0%)	19 (57.6%)	
Less than total	55 (39.0%)	14 (42.4%)	
CLND	141 (100%)	32 (97.0%)	0.190
LND	40 (28.4%)	9 (27.3%)	0.900
Thyroiditis	53 (37.6%)	10 (30.3%)	0.433
Pathology			0.572
PTC	138 (97.9%)	32 (97.0%)	
PDTC	3 (2.1%)	1 (3.0%)	
Tumor location			0.778
Upper	8 (5.7%)	3 (9.1%)	
Mid	113 (80.1%)	25 (75.8%)	
Lower	20 (14.2%)	5 (15.2%)	
Tumor size (cm)	2.0 ± 1.0 (range, 0.4–5.8)	2.2 ± 1.5 (range, 0.5–6.0)	0.476
Multifocality			0.138
Unilateral	24 (17.0%)	7 (21.2%)	
Bilateral	18 (12.8%)	7 (21.2%)	
Vascular invasion	12 (8.5%)	4 (12.1%)	0.509
Lymphatic invasion	98 (69.5%)	24 (72.7%)	0.716
Perineural invasion	8 (5.7%)	1 (3.0%)	1
Harvested LNs	25.9 ± 31.2 (range, 0–164)	25.7 ± 30.4 (range, 0–108)	0.978
Positive LNs	6.2 ± 7.7 (range, 0–33)	6.0 ± 6.2 (range, 0–22)	0.895
N stage			0.125
N0	40 (28.4%)	15 (45.5%)	
N1a	66 (46.8%)	10 (30.3%)	
N1b	35 (24.8%)	8 (24.2%)	
RAI ablation	62 (44.0%)	17 (51.5%)	0.433
RAI mean dose (mCi)	111.3 ± 46.5	117.6 ± 52.9	0.872
Recurrence	9 (6.4%)	1 (3.0%)	0.690

Data are expressed as patient counts (%) or means ± standard deviations. Abbreviations: CLND, central lymph node dissection; LND, lateral neck dissection; PTC, papillary thyroid carcinoma; PDTC, poorly differentiated thyroid carcinoma; LN, lymph node; N, node; RAI, radioactive iodine.

**Table 4:** Univariate and multivariate analyses of clinical factors influencing the misjudgment of extrathyroidal extension (Group A vs. Group B).

	Univariate		Multivariate	
	OR (CI)	*p*-Value	OR (CI)	*p*-Value
Age (years)	0.978 (0.955–1.002)	0.078	0.961 (0.932–0.990)	0.009
Male	0.841 (0.365–1.939)	0.685		
Palpation	0.711 (0.287–1.759)	0.460		
LND	0.673 (0.284–1.594)	0.368		
Thyroiditis	0.969 (0.461–2.034)	0.933		
Tumor location				
Upper	Ref.		Ref.	
Mid	0.165 (0.057–0.477)	0.001	0.182 (0.056–0.591)	0.005
Lower	0.356 (0.101–1.249)	0.107	0.337 (0.085–1.329)	0.120
Tumor size (cm)	0.532 (0.338–0.837)	0.006	0.647 (0.393–1.065)	0.087
Multifocality				
Unilateral	1.891 (0.815–4.386)	0.138		
Bilateral	0.688 (0.187–2.525)	0.572		
Lymphatic invasion	0.395 (0.190–0.820)	0.013	0.292 (0.118–0.719)	0.007
Harvested LNs	0.992 (0.978–1.007)	0.296		
Positive LNs	0.985 (0.936–1.036)	0.551		
N stage				
N0	Ref.			
N1a	0.839 (0.372–1.895)	0.673		
N1b	0.615 (0.221–1.715)	0.353		

Data are expressed as hazard ratios (HRs) and 95% confidence intervals (CIs). *p* < 0.05 was considered statistically significant. Abbreviations: LND, lateral neck dissection; LN, lymph node; N, node.

## Data Availability

The data presented in this study are available on request from the corresponding author.

## Supplementary Materials

The following supporting information can be downloaded at: https://www.mdpi.com/article/10.3390/cancers17243914/s1, Table S1: Clinical characteristics of patients with recurrent disease during follow-up.
