# Supplementary material for: Accuracy and Clinical Significance of Intraoperative Gross Extrathyroidal Extension (T3b) Assessment in Differentiated Thyroid Carcinoma
Source: Cancers (Basel). 2025 Dec 7;17(24):3914. doi: 10.3390/cancers17243914 (PMC12731079; doi:10.3390/cancers17243914)
Supplement: Supplementary file 1 [file cancers-17-03914-s001.zip › cancers-3994269-supplementary.pdf]

Supplementary Table S1. Clinical characteristics of patients with recurrent disease during follow-up

Group	Age/sex	Initial tumor	N stage	Time to recurrence (month)	Site of recurrence	Treatment for recurrence
A	47/M	PTC-3.3cm	N1a	7	Level VI LN (ipsilateral)	surgery
A	65/M	PTC-3.2cm	N1a	24	Level III&IV LN (ipsilateral)	surgery
A	57/M	PTC-2.6cm	N1b	23	op bed + Level III&VI LN (ipsilateral)	surgery + RAI + RFA
A	76/F	PTC-2.4cm	N1a	18	Level III&IV LN (ipsilateral)	RFA
A	22/M	PDTC-5.3cm	N1b	4	Level IV LN (bilateral)<br>Level VI LN, possibly invading posterior wall of trachea & esophagus (ipsilateral)	RTx + chemotherapy
A	22/F	PTC-4.5cm	N1b	20	Lung metastasis	observation
A	17/F	PTC-3.4cm	N1b	14	Level VI LN (ipsilateral)	RFA
A	71/M	PDTC-3.5cm	N0	7	Level II LN (ipsilateral)<br>Liver & bone metastasis	RTx + chemotherapy
A	30/M	PTC-1.7cm	N1b	12	Level VI LN (ipsilateral)	surgery + RAI
B	22/F	PTC-1.8cm	N1b	15	Level II LN (ipsilateral)	surgery + RAI
C	69/M	PTC-6.0cm	N1b	10	Level IV LN (ipsilateral)	surgery + RAI

Abbreviations : N, node; M, male; F, female; PTC, papillary thyroid carcinoma; PDTC, poorly differentiated thyroid carcinoma; LN, lymph node; RAI, radioactive iodine; RFA, radiofrequency ablation; RTx, radiation therapy
